# Mapping the stability of malaria hotspots in Bangladesh from 2013 to 2016

**DOI:** 10.1186/s12936-018-2405-3

**Published:** 2018-07-11

**Authors:** Andrés Noé, Sazid Ibna Zaman, Mosiqure Rahman, Anjan Kumar Saha, M. M. Aktaruzzaman, Richard James Maude

**Affiliations:** 1 Centre for Tropical Medicine and Global Health, Nuffield Department of Medicine Research Building, University of Oxford, Old Road Campus, Roosevelt Drive, Oxford, OX3 7FZ UK; 2 Mahidol-Oxford Tropical Medicine Research Unit, Faculty of Tropical Medicine, Mahidol University, 3/F, 60th Anniversary Chalermprakiat Building, 420/6 Rajvithi Road, Bangkok, 10400 Thailand; 3 National Malaria Elimination Programme, Directorate General of Health Services, Ministry of Health and Family Welfare, Dhaka, Bangladesh; 4 Harvard TH Chan School of Public Health, Harvard University, Boston, USA

**Keywords:** Hotspots, Spatial epidemiology, Heterogeneity, Spatiotemporal, Incidence, GIS, Cartography

## Abstract

**Background:**

Malaria claims hundreds of thousands of lives each year, most of them children. A “malaria-free world” is the World Health Organization’s vision, but elimination from the southeast Asian Region is hampered by factors including anti-malarial resistance and systematic underreporting. Malaria is a significant public health problem in Bangladesh and while there have been recent gains in control, there is large spatial and temporal heterogeneity in the disease burden. This study aims to determine the pattern and stability of malaria hotspots in Bangladesh with the end goal of informing intervention planning for elimination.

**Results:**

Malaria in Bangladesh exhibited highly seasonal, hypoendemic transmission in geographic hotspots, which remained conserved over time. The southeast areas of the Chittagong Hill Tracts were identified as malaria hotspots for all 4 years examined. Similarly, areas in Sunamganj and Netrakona districts in the Northeast were hotspots for 2013–2016. Highly stable hotspots from 1 year predicted the following year’s hotspot locations in the southeast of Bangladesh. Hotspots did not appear to act as sources of spread with no evidence of consistent patterns of contiguous spread or recession of hotspots as high or low transmission seasons progressed.

**Conclusions:**

Areas were identified with temporal and spatial clustering of high malaria incidence in Bangladesh. Further studies are required to understand the vector, sociodemographic and disease dynamics within these hotspots. Given the low caseloads occurring in the low transmission seasons, and the conserved nature of malaria hotspots, directing resources towards these areas may be an efficient way to achieve malaria elimination in Bangladesh.

## Background

**Fig. 1:**
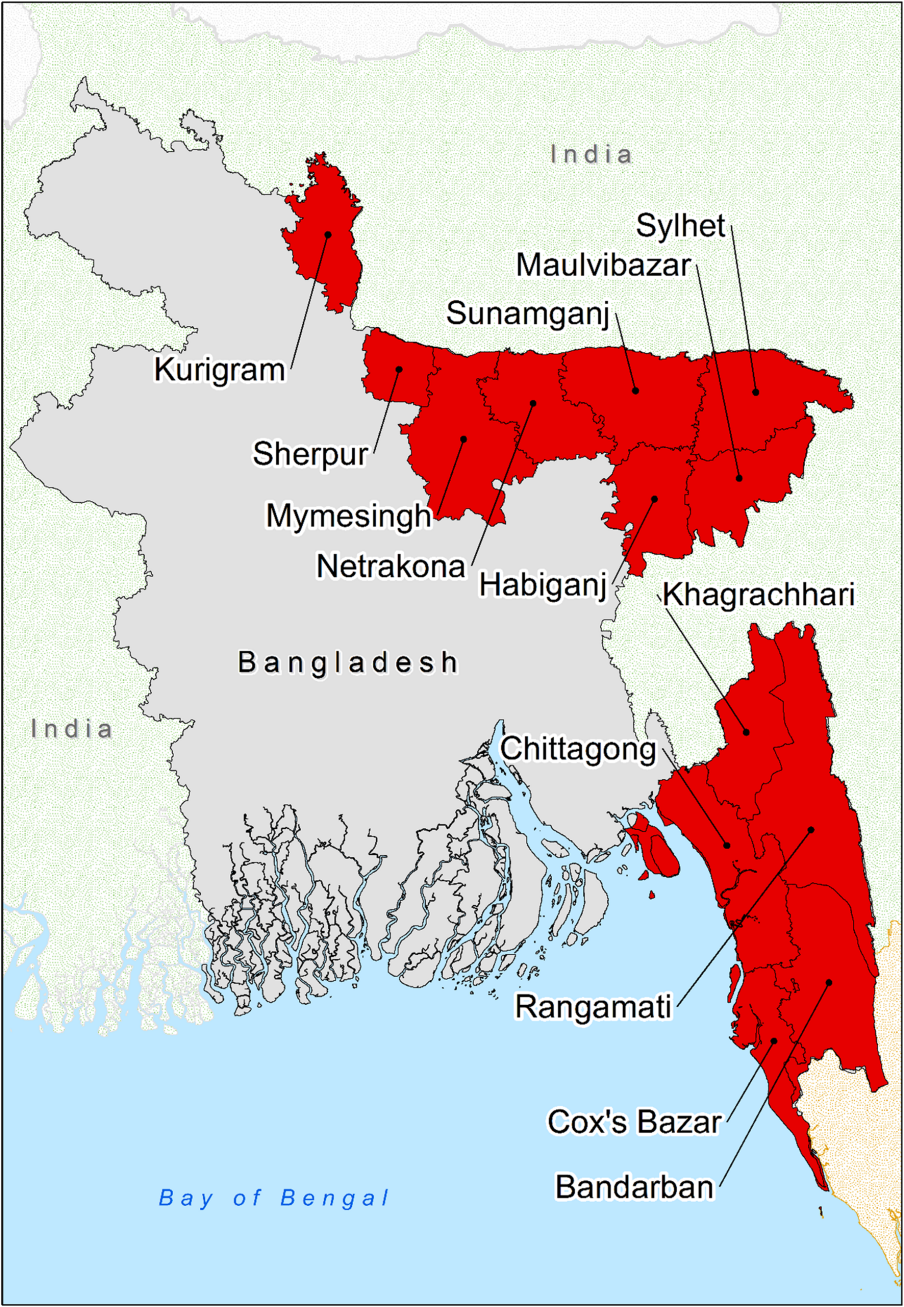
The 13 malaria-endemic districts of Bangladesh. SeA is the area highlighted red to the southeast of Bangladesh (Bandarban, Chittagong, Cox’s Bazar, Khagrachhari and Rangamati) and NeA is the eight districts highlighted red to the northeast of Bangladesh (Habiganj, Kurigram, Maulvibazar, Mymesingh, Netrakona, Sherpur, Sunamganj and Sylhet). Note: the CHTs are Khagrachhari, Rangamati and Bandarban. CHTs, Chittagong Hill Tract districts. *NeA* The Northeast Area, *SeA* The Southeast Area

Malaria is a significant public health problem in Bangladesh. The disease is endemic in 13 of 64 districts, with over 17 million people at risk (over 8% of the total population) [1, 2]. Cox’s Bazar and the Chittagong Hill Tract districts (CHTs) (Bandarban, Khagrachhari and Rangamati) report over 90% of cases and 80% of deaths due to the disease (Fig. 1) [3]. Clinical cases of malaria cluster seasonally in Bangladesh. Over 80% of cases occur in the high transmission period from May to October, when there is increased rainfall and high humidity [4]. Eighty-eight percent of cases have *Plasmodium falciparum* on microscopy, with the majority of the remaining cases reported to be caused by *Plasmodium vivax* [2, 5]. A renewed intensity of malaria control efforts in Bangladesh has seen malaria incidence and mortality rates drop by over 75% from 2010 to 2015 [2, 6].

A coalition of governmental and Non-Governmental Organizations (NGOs) lead the malaria elimination efforts within Bangladesh. The National Malaria Elimination Programme (NMEP) works with a coalition of 16 NGOs steered by BRAC (formerly Building Resources Across Communities, now BRAC), delivering a variety of interrelated community-wide intervention programmes [3] The BRAC-Led NGO Consortium (BLNC) operates in non-urban areas, has grass-roots connections with local populations and implements most malaria control activities, under the direction of the NMEP [7]. Haque et al. [1] performed an epidemiologic and economic assessment of the NMEP’s functions over 2008–2012, during which malaria prevalence, severe disease and mortality all decreased. These reductions coincided with the initiation of intensified malaria interventions and were associated with increased LLIN coverage [1]. The NMEP’s successes have heralded calls for malaria elimination in Bangladesh.

Available data on the distribution of malaria in Bangladesh are incomplete which impede planning and allocation of resources for elimination. Total confirmed cases in the public sector (government and BLNC) vary from around 25,000–50,000 per year [3, 5], but many are thought to not receive confirmatory testing [8]. Incidence rates from teaching, specialty and NGO hospitals, and private clinics were not included in nationwide estimates, although this is now changing [9]. These limitations of available data in Bangladesh compound the effect that heterogeneous malaria transmission has on control efforts.

Heterogeneity refers to the situation whereby “infection and disease are concentrated in a small proportion of individuals and not distributed evenly across the population [10].” Heterogeneity in transmission levels of an infectious disease are thought to negatively impact control efforts as it may be ineffective to use the same strategy across a large heterogeneous area [11, 12]. Geographic variations in malaria transmission and incidence rate have been reported down to the household level and even in locations that have relatively uniform geographical characteristics [4, 13, 14]. Malaria can be clustered in time, as well as space. Ahmed et al. [4] demonstrated that symptomatic *P. falciparum* malaria maintains a pattern of seasonality in hypoendemic districts of Bangladesh. The authors estimated that hotspots encompassing a third of the total population in these areas accounted for 80% of symptomatic malaria [4]. It is thought that strengthened control in geographic hotspots is imperative to efficiently achieving malaria elimination [4, 12, 14, 15]. Additionally, reducing transmission in hotspots may reduce transmission in the wider community [12, 16], may be a more cost-effective means to approach elimination [14], and may ethically justify “unequal, but equitable,” allocation of resources [17]. There have been no studies that have investigated the spatial heterogeneity of malaria over significant periods of time, in both endemic areas and to a fine spatial scale in Bangladesh. To address the gaps in existing knowledge, statistically significant malaria hot spots were mapped over time and the predictive capability of the clusters was assessed. The end goal of this work was to inform the planning of interventions to be carried out by the NMEP of Bangladesh.

## Methods

### Study area

**Fig. 2:**
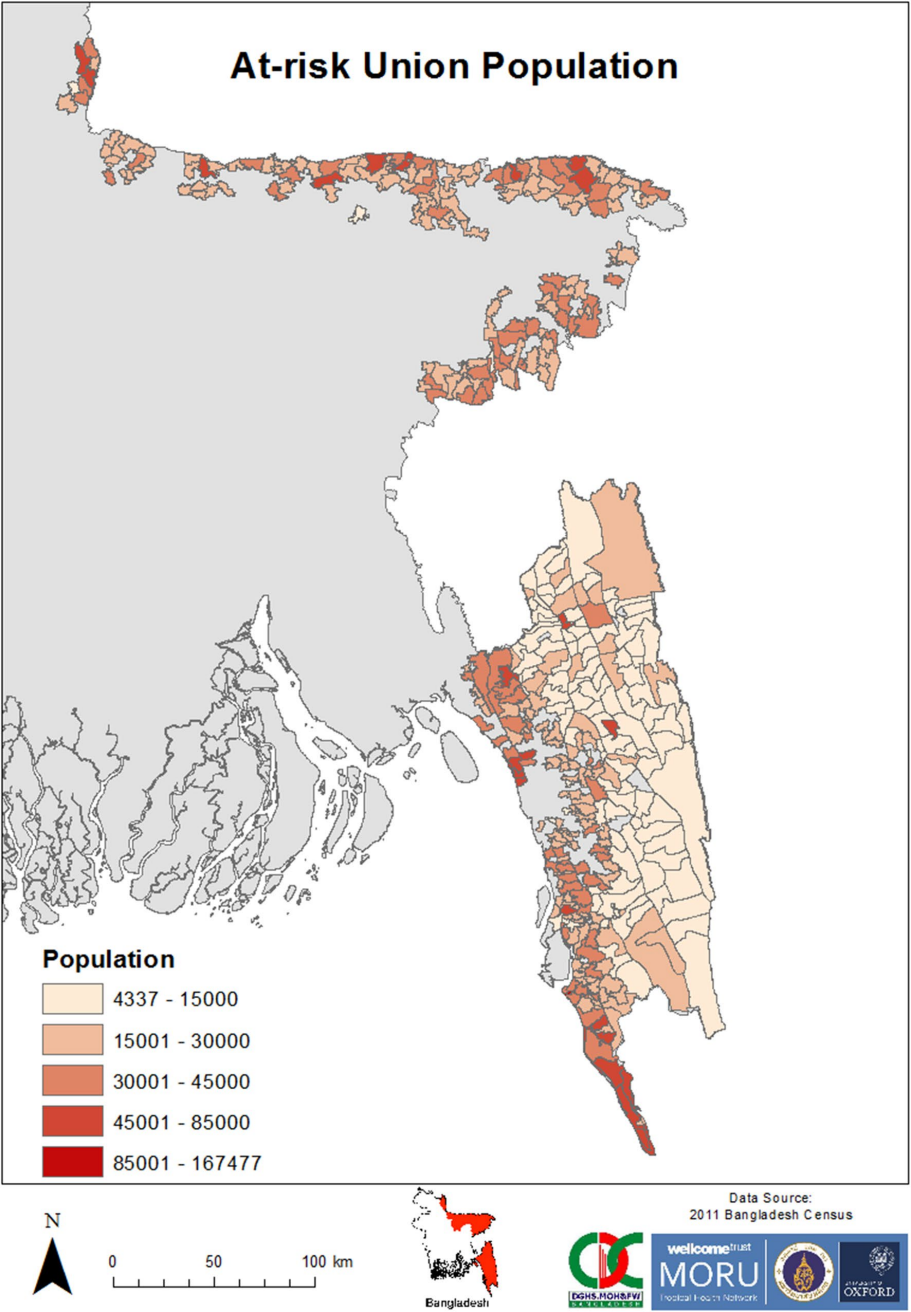
Population counts in the malaria at-risk unions in Bangladesh from 2013 to 2016

**Fig. 3:**
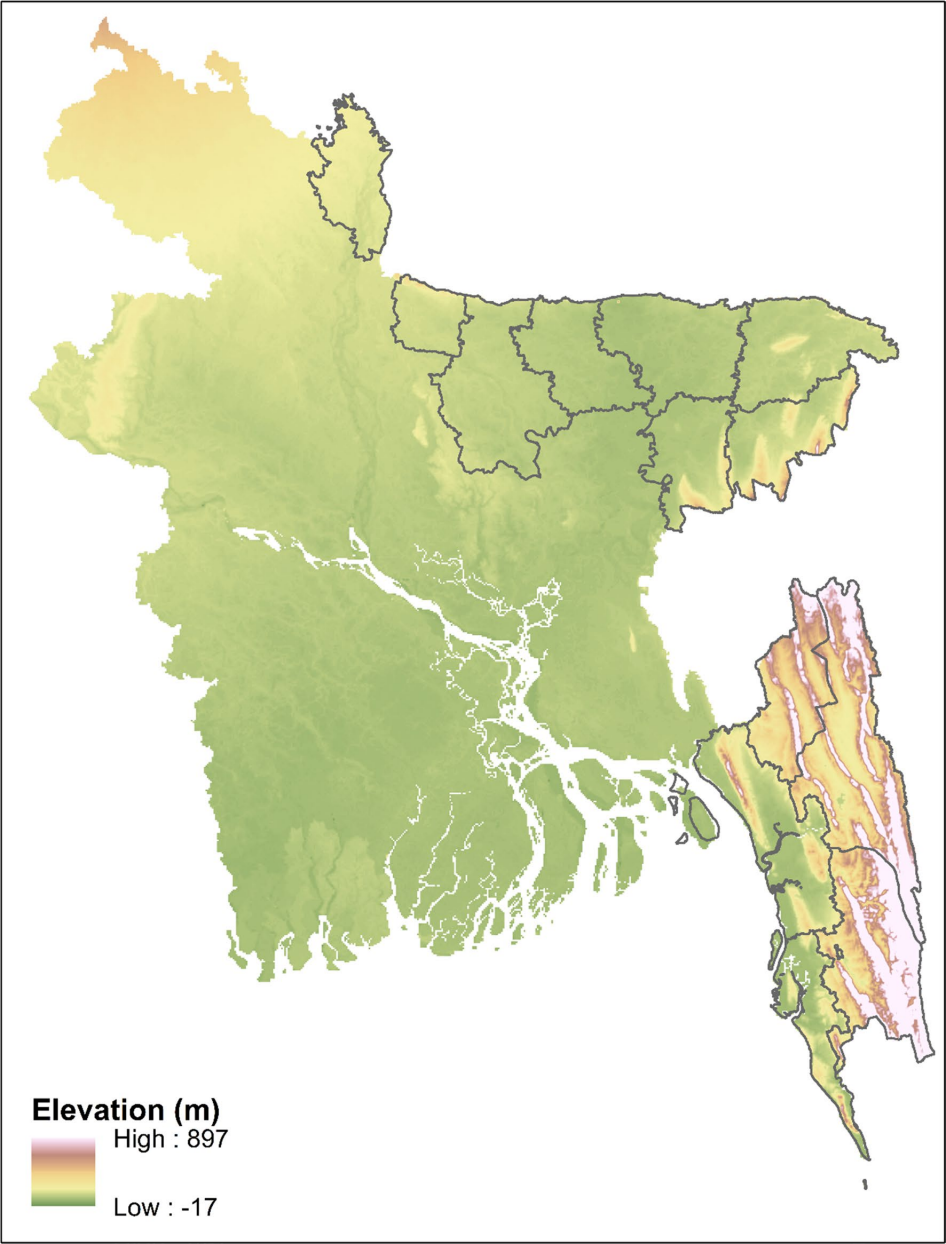
Elevation of land in Bangladesh. The 13 endemic districts are outlined. Data source: diva-gis.org

Bangladesh is partitioned into 7 divisions, 64 districts, 485 upazilas and 4498 unions, as of 2014. Unions are geographically and demographically heterogeneous governance sub-units [18]. Eight of the thirteen malaria-endemic districts are in the northeast and share borders with India, while the other five districts are in the southeast, bordering India and Myanmar (Fig. 1). Two geographic areas of Bangladesh are defined in this paper. “The Northeast Area” (NeA) refers to the eight malaria-endemic districts in the northeast, while “The Southeast Area” (SeA) refers to the five malaria-endemic districts in the southeast. These areas vary in terms of population, topography and population at risk of malaria (Figs. 1, 2, 3 and Table 1). Malaria incidence has historically been highest in SeA, namely the CHTs and Cox’s Bazar [3]. These areas are less accessible and less developed due to elevated and forested terrain (Tables 1 and 2). The SeA is populated by numerous groups of distinct minority ethnic groups, has historically had the most highly endemic areas and, compared to NeA, contains districts with relatively low population density (except Chittagong) [9, 18, 19]. By contrast, NeA is home to a higher proportion of the majority Bengali ethnic group and has been referred to as the ‘pre-elimination area’ due to its relatively low incidence of malaria [3].

**Table 1:** Characteristics of SeA and NeA

	Southeast area (SeA)	Northeast area (NeA)
Area	19,901 km^2^	23,153 km^2^
Borders	North: India (Tripura) East: India (Mizoram), Myanmar (Chin) South: Myanmar (Rakhine) West: Bay of Bengal	North: India (Meghalaya) East: India (Assam) South: India (Tripura), central Bangladesh West: Central/West Bangladesh
Number of districts	5	8
Number of upazilas (number of malaria-endemic upazilas)	47 (44)	71 (27)
Number of unions with at least one reported case of malaria between 2013 and 2016 (% of total unions in the area)	259 (50.3%)	153 (19.9%)
Population at risk of malaria within these unions (% of total population in the area)	5,531,321 (48.6%)	4,274,924 (20.9%)
Median union population at risk of malaria (IQR)	18,009 (17,341)	27,178 (11,630)
Malaria ecotypes	Forested hills and forest fringes, two major urban centres (Chittagong city and Cox’s Bazaar)	Forest fringes and plains, two major urban centres (Sylhet and Mymensingh)

The BRAC micro-stratified dataset was used to calculate the number of unions in which there was malaria between 2013 and 2016. It is important to note that this is likely an underestimate of the total unions in SeA and NeA with malaria, as the BLNC is not the only organization that collects malaria data (e.g. the government health workers also collect surveillance data). The population at risk was calculated by summing the population of each union that reported a case of malaria (in the BRAC micro-stratified dataset) from 2013 to 2016

**Table 2:** Average annual incidence in each area considered

Area	2013 incidence (per 1000)	2014 incidence (per 1000)	2015 incidence (per 1000)	2016 incidence (per 1000)
13 Endemic districts	1.70	3.90	2.78	2.08
SeA	2.78	6.81	4.88	3.66
NeA	0.297	0.134	0.0678	0.0430

Confirmed malaria cases per 1000 population at risk

### Data source

The NMEP and BLNC operate parallel malaria reporting systems that report cases mostly from separate areas. The BLNC collects and manages approximately 80% of the malaria surveillance data in Bangladesh and operates primarily in non-urban settings that are often remote (unpublished data, NMEP) with reporting at union level. Contrastingly, the NMEP mainly records cases in urban and more developed areas with reporting at upazila level. As this analysis was at union level only data from BLNC were used. Over 81% of the total positive cases in Bangladesh are diagnosed in the community with Rapid Diagnostic Tests (RDTs) by BLNC-employed community health workers/volunteers (unpublished data, NMEP). Monthly union level data from BLNC are the highest spatial and temporal resolution data available on malaria incidence in Bangladesh. The data available for this study did not include information on parasite species or patient demographics.

### Ethical approval

This study analysed only anonymized, aggregated secondary data thus ethical approval was not required.

### Malaria diagnosis

In 2013 and 2014, the First Response Malaria pLDH/HRP Combo Card Test (Premier Medical Corporation Ltd., Nani Daman, India) was used by BLNC. In mid-2015, there was a transition to the ONE STEP Malaria HRP2 (*P. falciparum*)/pLDH (Pan) Antigen RDT (Standard Diagnostics Inc., Gyeonggi-do, Republic of Korea). These two RDTs were used for the detection of *P. falciparum*, non-*P. falciparum* species and mixed infections. In August 2016, the CareStart Malaria *P. falciparum*/*P. vivax* (HRP2/pLDH) Combo RDT (AccessBio Inc., Somerset, USA) was implemented as the RDT of choice, which was capable of detecting *P. falciparum*, *P. vivax* and mixed infections. Information was not available on how quickly the new RDT was rolled out.

### Analytical methods

The “population at risk” of malaria was calculated for an area by summing the populations of all unions (with data from the 2011 nationwide census) that had at least one case of malaria between 2013 and 2016. The population at risk was used to calculate incidence for each union. By adding geographic codes to the incidence dataset, the incidence data for 412 unions were matched to the corresponding polygons in an administrative unit shapefile using ArcGIS 10.4.1 (ESRI Inc., Redlands, CA) to generate malaria incidence maps for 2013–2016.

The Getis-Ord Gi* statistic [20] employed by the Hotspot Analysis tool in ArcGIS 10.4.1 was used to analyse the incidence maps. This statistic indicates whether features with high or low values cluster spatially more than what would be expected by chance. It determines whether the difference between the local mean (e.g. the disease incidence in a union—the “Feature”—and its nearest unions—the “Neighbourhood”) is significantly different from the overall mean (the incidence in all unions within the region—the “Study Area”). That is, the statistic compares the local sum of a Feature and Neighbourhood to the sum of all features in the Study Area. The GOGi* results in a Z-score for each feature that identifies clusters of high incidence (“hotspots”) if significantly positive (greater than 1.96 for a 95% confidence interval (95% CI)). The settings used for the Hotspot Analysis tool were contiguity (edges and borders) and Euclidean distances.

**Fig. 4:**
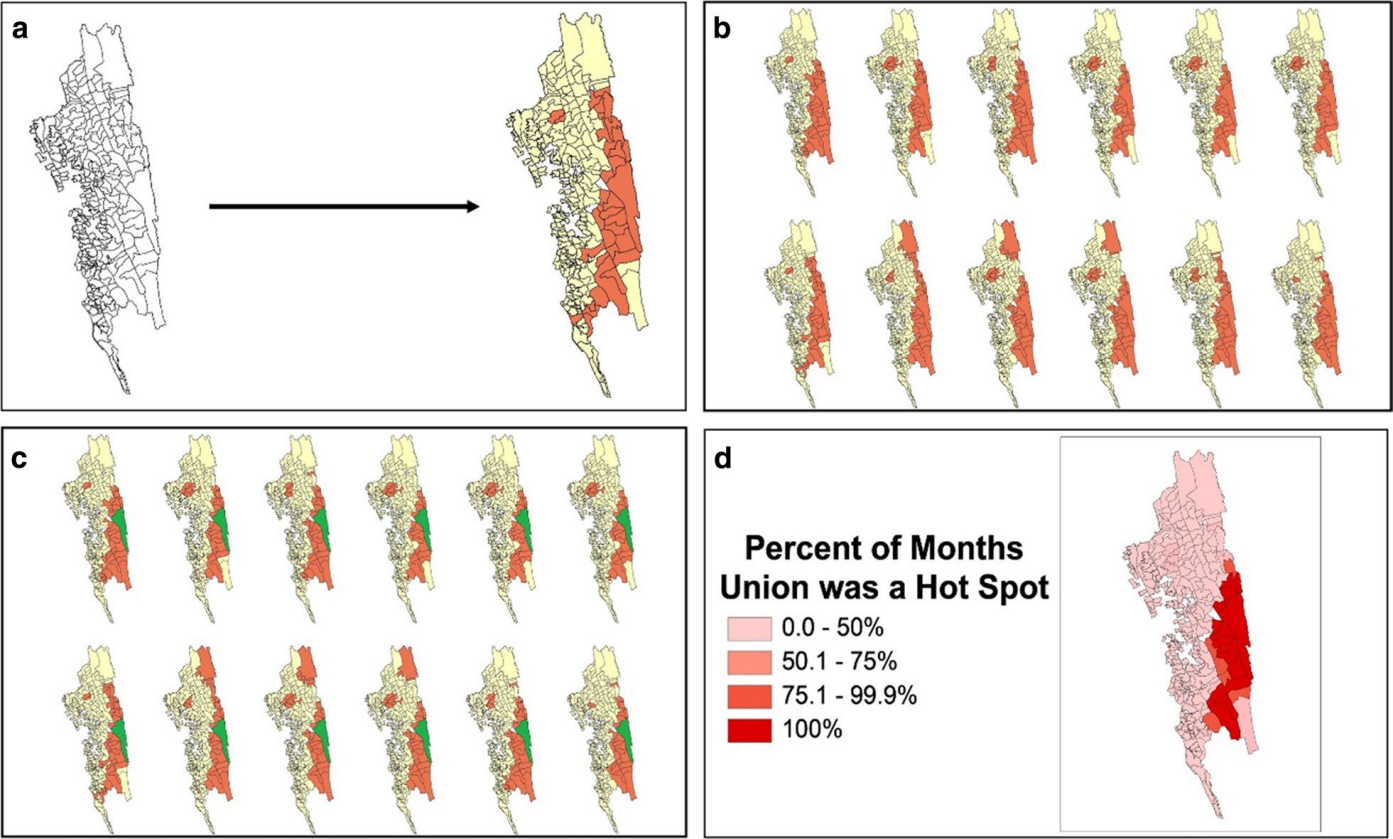
Depiction of the stability map production process. The four panels show an example of how a stability map was produced from monthly incidence data for 1 year. **a** Hotspot analysis for 1 month. The Getis-Ord Gi* statistical technique identifies hotspots of malaria incidence to a 95% confidence level (marked in orange). The features that are not statistically significant hotspots are marked in yellow. **b** Monthly hotspots for each month in the year. The Getis-Ord Gi* technique identifies hotspots for each month of the year in question. **c** Hotspot stability identification. Each geographical area is examined for how many months it was identified to be a hotspot in the year. For example, Farua, the area highlighted in green, was a hotspot for all 12 months of the year. Therefore, the percent of months the union was a Hotspot is 100%. **d** Stability map depiction. The amount of time each geographic area was a hotspot is displayed on a map. Farua can be seen shaded in the deepest red, indicating it was a hotspot for 100% of that year

Monthly hotspot maps were generated. Their results were compiled to stratify unions into the percentage of months each was a hotspot as a measure to indicate hotspot stability (Fig. 4). This process was performed for 2013–2016, each year and each season (comparing high transmission season (June–September) to low transmission season (October to May)). Using these percentages, malaria hotspot stability maps were created. These maps portray the percentage of months that a union was a hotspot. By visualizing the progression of individual hotspot maps over time an assessment was made as to whether hotspots acted as sources of spread of malaria across union boundaries.

## Results

The total number of malaria cases reported by BLNC between 2013 and 2016 was 102,582; with 16,631 occurring in 2013, 38,229 in 2014, 27,276 in 2015 and 20,446 in 2016. From 2013 to 2016, June to September were the 4 months with the most cases of malaria. In 2013, 8907 cases (53.56% of 2013’s cases) occurred between June and September; in 2014, 26,113 cases (68.31%); in 2015, 15,423 (56.54%) and in 2016, 13,249 (64.80%).

Using the at-risk population within malaria-endemic unions (9,806,245 people, Table 1), the average yearly incidence was 2.62 malaria cases per 1000 population at risk (“per 1000”). The nationwide incidence was greatest in 2014 (Table 1). The average annual incidence between 2013 and 2016 was 4.53 and 0.135 cases per 1000 in SeA and NeA, respectively.

**Fig. 5:**
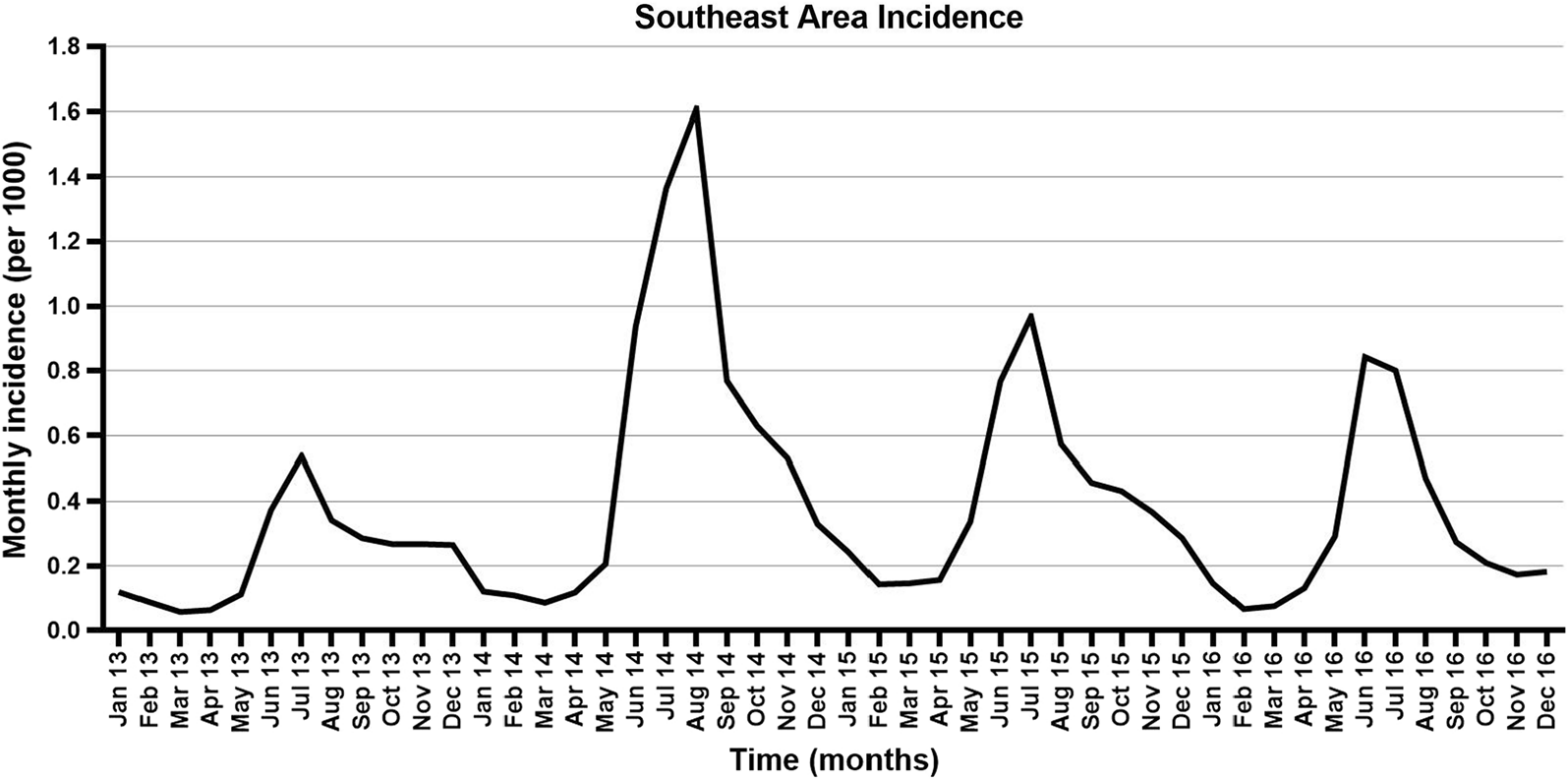
Monthly malaria incidence in SeA as reported by BLNC. Malaria diagnoses increased significantly throughout 2014 compared to 2013. The following 2 years had smaller peaks in the high transmission season. “Per 1000” = malaria cases per 1000 population at risk

When compared to other years, 2014 had the most cases reported for each month (Fig. 5). Incidence declined throughout 2013–2016 in NeA, with peaks and troughs occurring at different times and with decreasing magnitudes each year. SeA accounted for 100,265 (97.74%) total malaria cases from 2013 to 2016, comprising: 15,362 (92.37%) in 2013; 37,655 (98.50%) in 2014; 26,986 (98.94%) in 2015; and 20,262 (99.10%) in 2016. From 2013 to 2016 in SeA, 10.9% of the at-risk population accounted for 80.1% of malaria cases; in NeA, 32.6% accounted for 80.3% of cases.

The 10 unions with the highest average annual incidence (2013–2016) were all within SeA, with five bordering India and/or Myanmar and nine clustered in Bandarban and Rangamati (Table 3). Among the 10 unions with the highest average incidence within NeA, eight bordered India and six had an incidence of less than one per 1000 people (Table 3).

**Table 3:** The 10 unions with the highest average annual incidence, by study area from 2013 to 2016

	#1	#2	#3	#4	#5	#6	#7	#8	#9	#10
SeA										
Incidence	127	123	107	97.7	78.5	72.9	71.9	69.9	69.6	68.0
Union	Farua	Remakry	Thanchi	Dumdumya	Tindu	Alikhong	Nowa Patang	Alikadam	Lama	Dulyatali
District	R	B	B	R	B	B	B	B	B	K
NeA										
Incidence	1.97	1.56	1.11	1.03	0.843	0.558	0.547	0.486	0.424	0.360
Union	Rangchhati	Rangar Char	Durgapur	Uttar Bangshikunda	Ranikhai Uttar	Lakshmansree	Mohanpur	Uttar Badal	Rajghat	Uttar Sreepur
District	N	Su	N	Su	Sy	Su	Su	Su	M	Su

The average annual number of confirmed cases per 1000 population at risk is the reported incidence. This table was created using BRAC micro-stratified data with spatial resolution down to union-level

*B* Bandarban, *K* Khagrachhari, *M* Maulvibazar, *N* Netrakona. *R* Rangamati, *Su* Sunamganj, *Sy* Sylhet

**Fig. 6:**
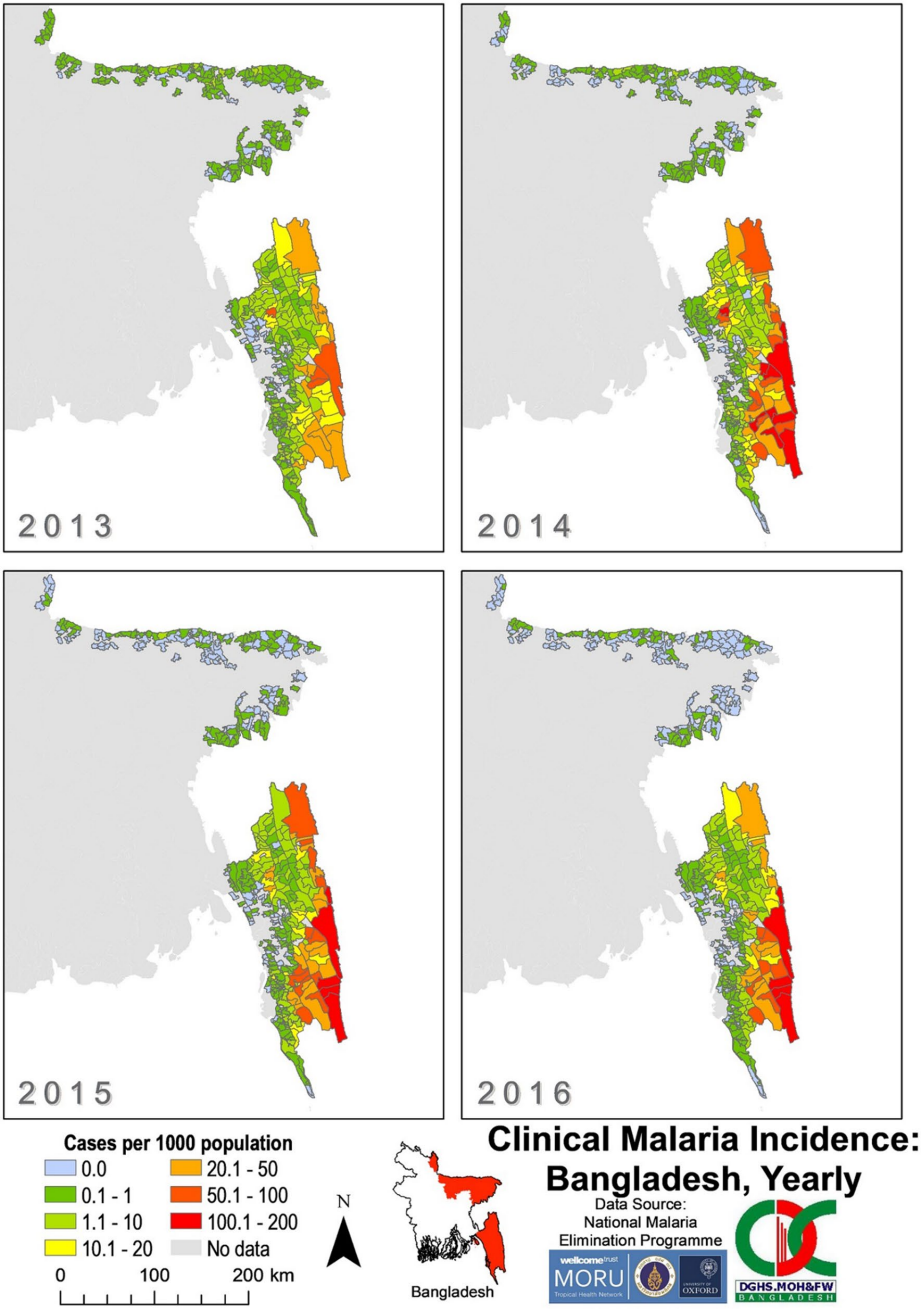
Clinical malaria incidence in Bangladesh by union from 2013 to 2016 as reported by BLNC. The “No data” category indicates that for that area, no cases of malaria were reported in the dataset

**Fig. 7:**
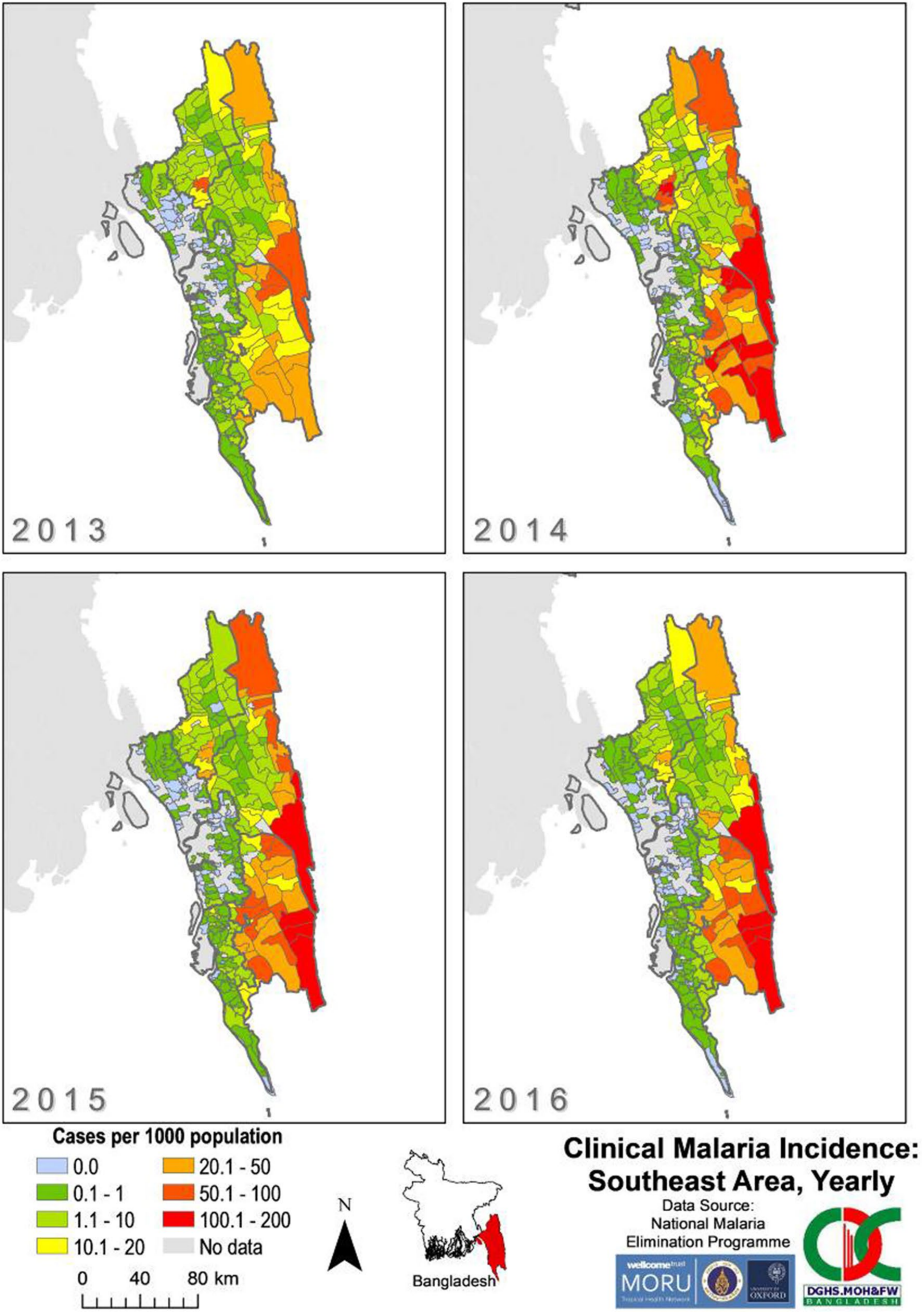
Clinical malaria incidence by union in SeA from 2013 to 2016. The “No data” category indicates that for that area, no cases of malaria were reported in the dataset. The dark grey outlines overlying the maps denote endemic district boundaries. Refer to Fig. 1 for an endemic district reference map

The overall incidence of malaria across Bangladesh was greater in SeA (median 0.713 cases/1000 population at risk/year) compared to NeA (median 0.0520 cases/1000 population at risk/year) for each year from 2013 to 2016 (Fig. 6). The number of unions with an incidence of 100–200 cases per 1000 increased from zero in 2013 to a peak of 10 in 2014 in SeA (Fig. 7). From 2014 to 2016, unions with incidences of 50–200 cases per 1000 became more concentrated in the southeast of SeA (bordering Myanmar and India) compared to 2013. From 2014 to 2016, there was an increase in the number of unions with an incidence of zero in SeA, mostly within Chittagong.

**Fig. 8:**
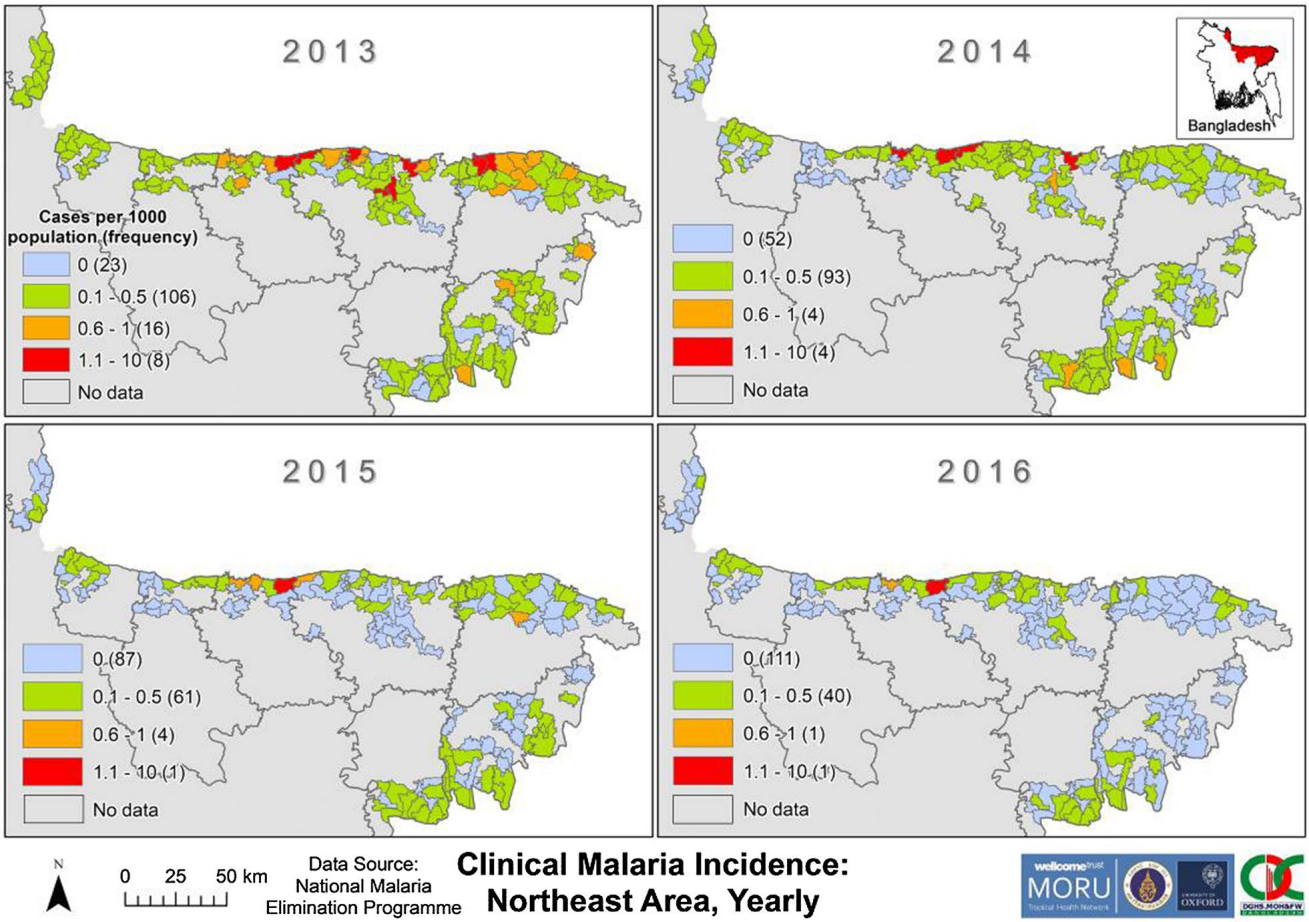
Clinical malaria incidence by union in NeA from 2013 to 2016. The “No data” category indicates that for that area no cases of malaria were reported in the dataset. The dark grey outlines overlying the maps denote endemic district boundaries. Refer to Fig. 1 for an endemic district reference map

From 2013 to 2016, the annual malaria incidence in all unions of NeA was less than 10 cases per 1000 (Fig. 8). Incidence declined in all unions and the number of unions with an incidence of zero increased throughout 2013–2016 in NeA. In 2013, high incidence unions in NeA were scattered throughout Netrakona, Sunamganj, Sylhet and Maulvibazar. By 2016, only two unions (Durgapur and Rangchhati—both bordering India) had incidences above 0.5 per 1000.

**Fig. 9:**
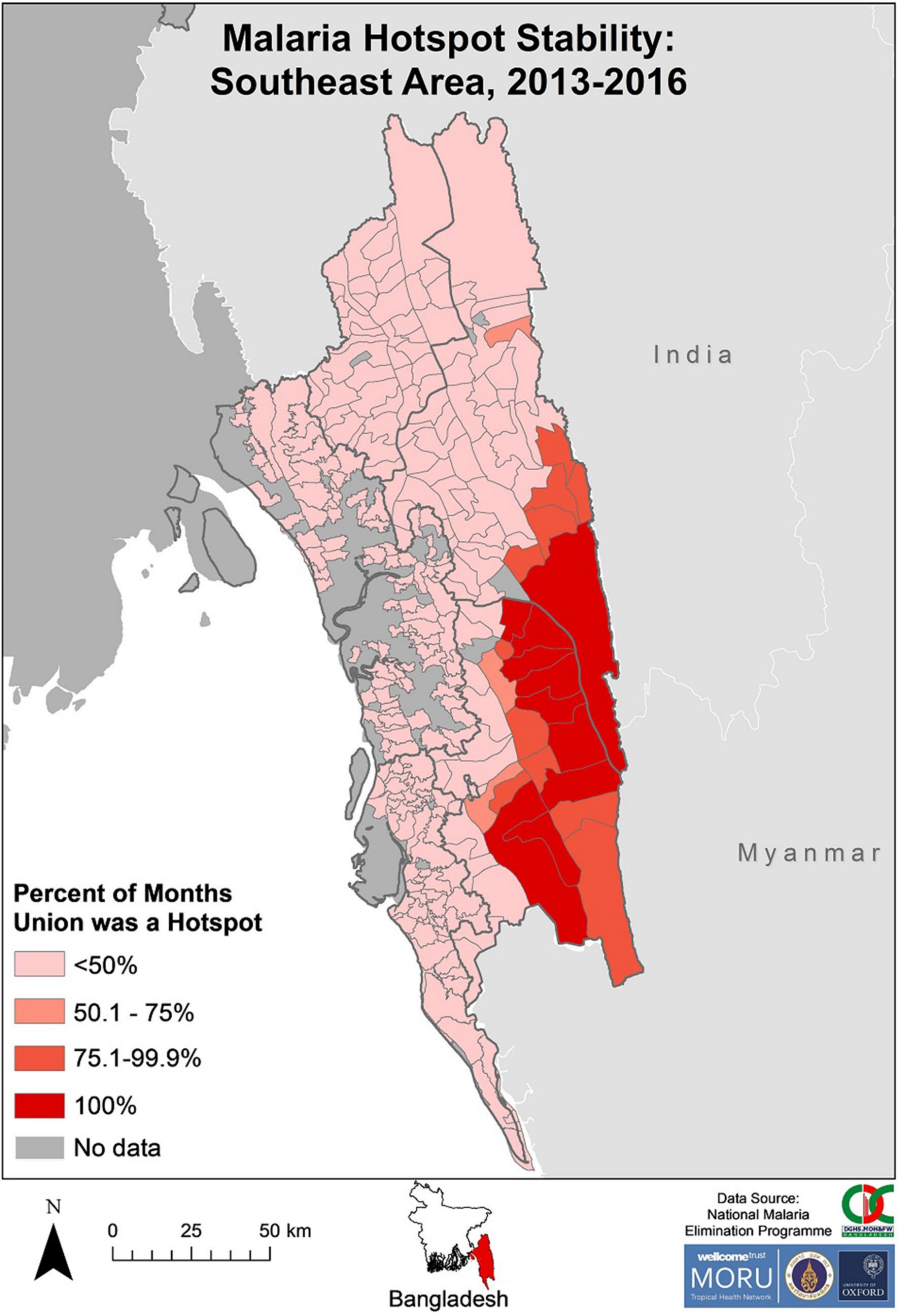
Malaria hotspot stability in SeA from 2013 to 2016. The “No data” category indicates that for that area no cases of malaria were reported in the dataset. The dark grey outlines overlying the map denote endemic district boundaries

There was clustering of SeA stable hotspots from 2013 to 2016 in the eastern areas of Rangamati and Bandarban districts (Fig. 9). The unions that were hotspots for 100% of 2013–2016 were contiguous and all except one were located on a district (Rangamati–Bandarban) and/or international border. All unions that were hotspots for > 50% of the time were contiguous except for one (located in the northern area of Rangamati). Unions within Khagrachhari, Chittagong and Cox’s Bazar were hotspots for ≤ 50% of the time.

**Fig. 10:**
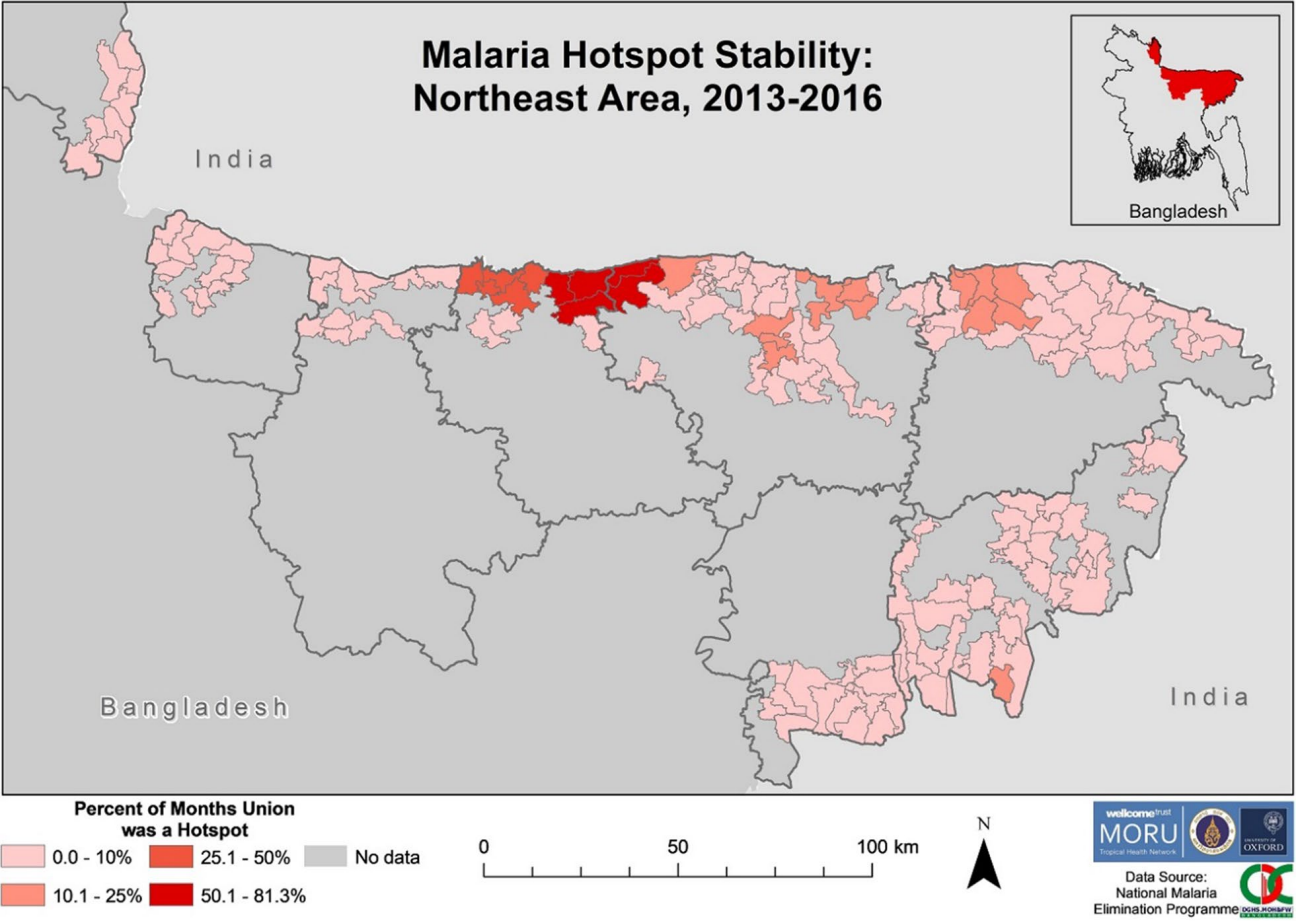
Malaria hotspot stability in NeA from 2013 to 2016. The “No data” category indicates that for that area no cases of malaria were reported in the dataset. The dark grey outlines overlying the maps denote endemic district boundaries

Hotspots were stable for a lower proportion of time in NeA (Fig. 10), when compared to SeA (Fig. 9). The five unions that were hotspots for > 50% of the time from 2013 to 2016 were all located on a district (Netrakona–Sunamganj) and/or international border. Netrakona had a proportionally higher number of unions with hotspot stability.

**Fig. 11:**
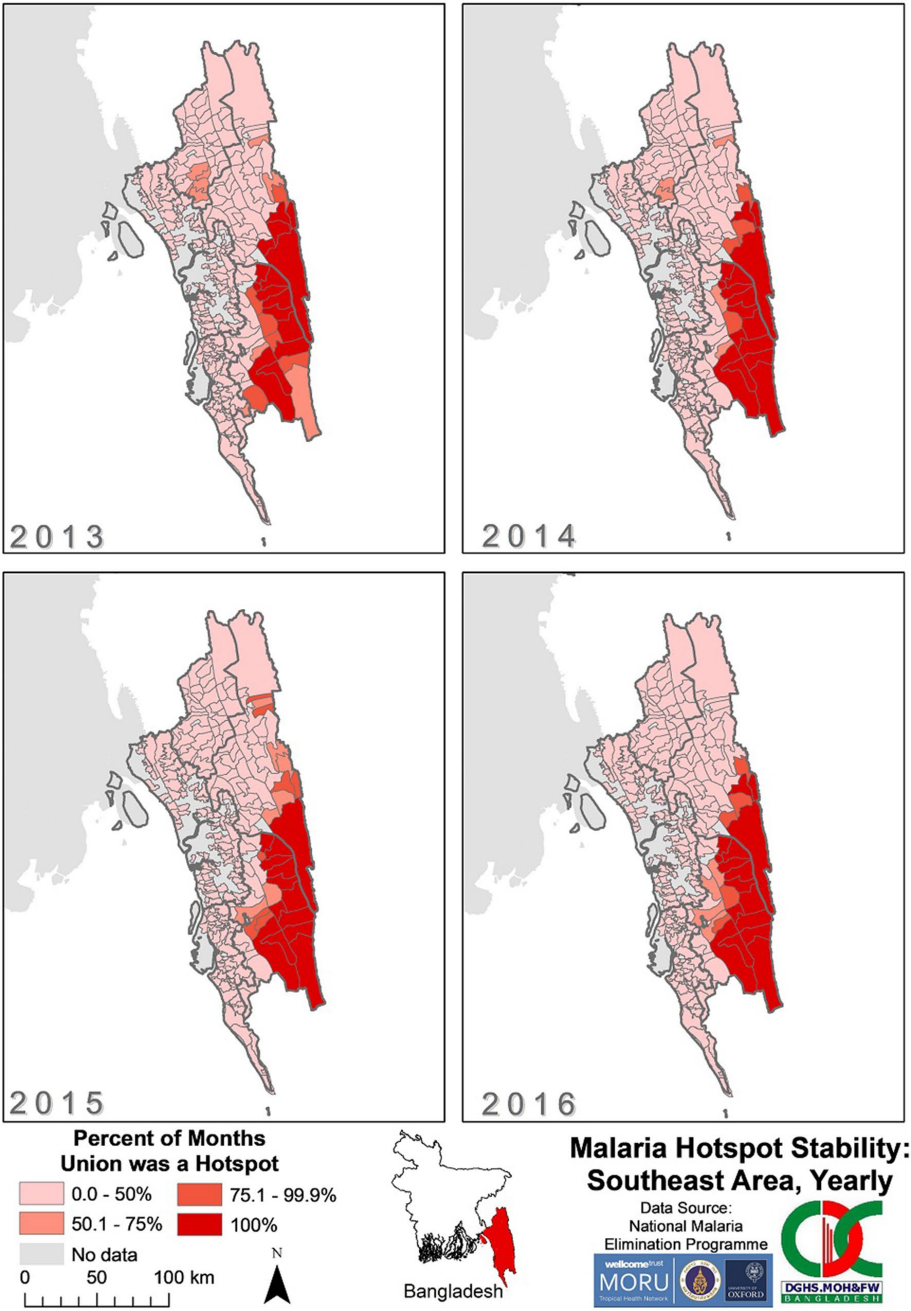
Malaria hotspot stability in SeA by year from 2013 to 2016. The “No data” category indicates that for that area no cases of malaria were reported in the dataset. The dark grey outlines overlying the maps denote endemic district boundaries

There was little movement of stable hotspots from year to year in SeA (Fig. 11). The unions in eastern Bandarban and southern Rangamati remained hotspots for 100% of each year from 2013 to 2016. The unions that were hotspots for 50–75% of each year from 2013 to 2015, located in Khagrachhari and northern Rangamati, were no longer stable hotspots in 2016. By 2016, all unions that were stable for > 50% of the time were located in eastern Bandarban and southern Rangamati.

**Fig. 12:**
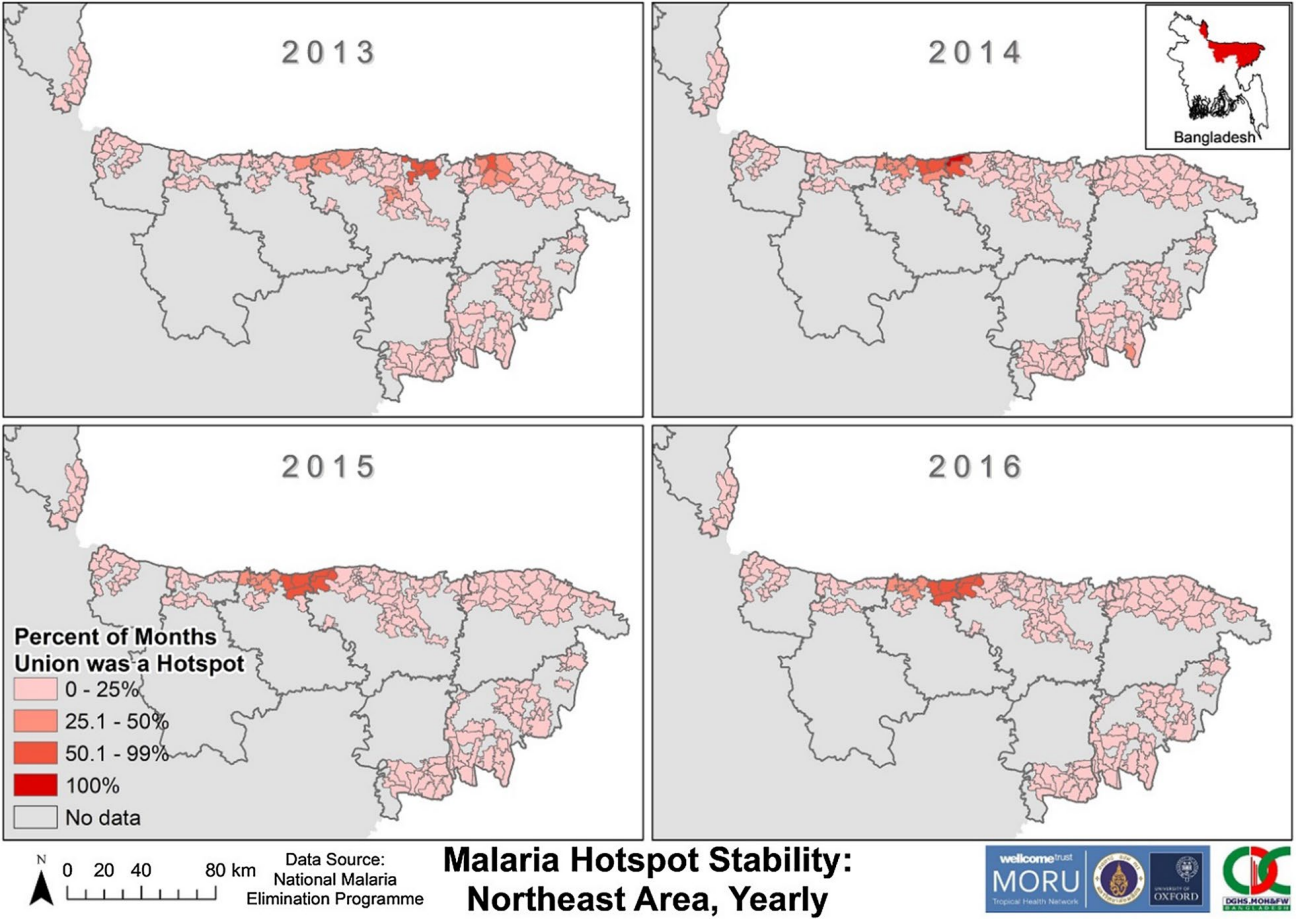
Malaria Hotspot Stability in NeA by year from 2013 to 2016. The “No data” category indicates that for that area no cases of malaria were reported in the dataset. The dark grey outlines overlying the maps denote endemic district boundaries

The unions that were hotspots for > 25% of 2013 in NeA were found throughout Netrakona, Sunamganj and Sylhet (Fig. 12). From 2014 to 2016 there was a cluster of unions that remained hotspots for > 25% each year in northern Netrakona and northwest Sunamganj. A subset of this cluster were hotspots for > 50% each year and were on a district (Netrakona-Sunamganj) and/or international border. One union (Uttar Bangshikunda) in Sunamganj was a hotspot for 100% of 2014.

**Fig. 13:**
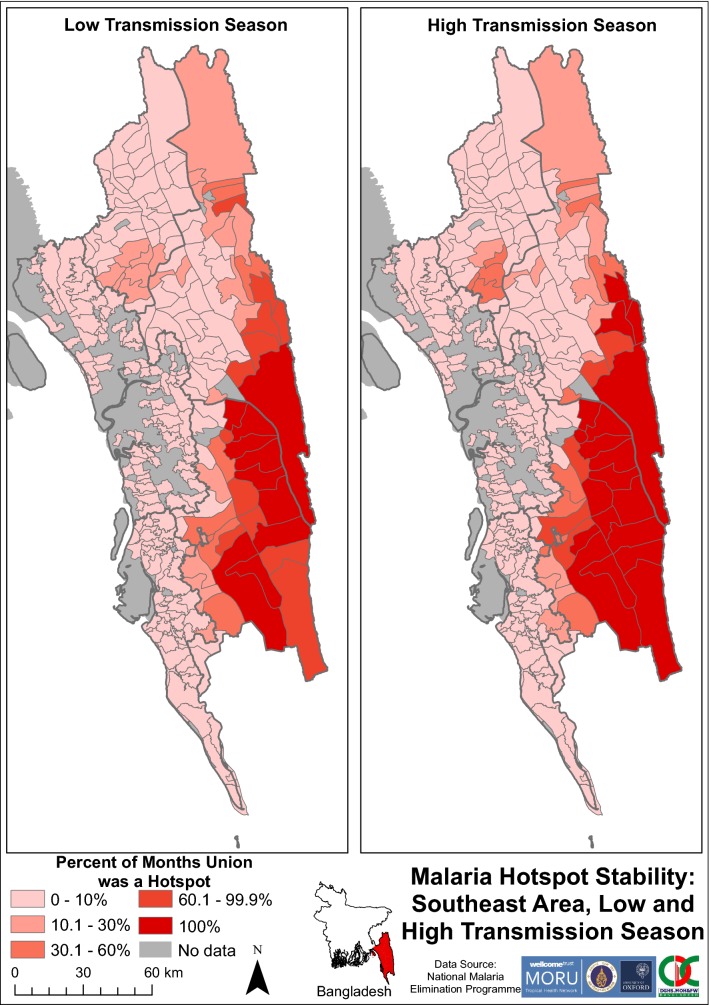
Malaria hotspot stability in SeA by transmission seasons. The “No data” category indicates that for that area no cases of malaria were reported in the dataset. The dark grey outlines overlying the maps denote endemic district boundaries

Hotspots remained consistent when comparing high and low transmission seasons (Fig. 13). Most unions in Bandarban and southeast Rangamati remained equally stable between 60 and 100% of both low and high transmission seasons. In the high transmission season, there was a concentrated cluster of unions with relatively high stability in Khagrachhari. During the low transmission season, this cluster exhibited lower hotspot stability.

There were several spatial clusters of statistically significant increased incidence in SeA when examining the monthly union hotspots. At the end of the 2013 malaria season and calendar year, the hotspots of SeA were spread across the eastern border of Khagrachhari, Rangamati and Bandarban. By contrast, in 2016, hotspots were relatively concentrated in the southeast of Rangamati, and Bandarban (compared with December of 2013 and 2016). In 2013 and 2014, there appeared to be contiguous outward spread of hotspots from within Khagrachhari from May to October. In 2015 and 2016, however, there were no hotspots identified during the same period in Khagrachhari. In 2015, from May to September, there was contiguous spread of hotspots from central Rangamati northwards to the northernmost tip of SeA and from Bandarban southwards into Chittagong and Cox’s Bazar. There were no consistent patterns of spread of malaria from hotspots in multiple years.

## Discussion

The main findings of this study were that from 2013 to 2016 in rural Bangladesh (1) cases of clinical malaria were clustered, both in space and time, (2) hotspots were stable between 2013 and 2016 and (3) hotspots did not consistently act as sources of spread.

The average annual malaria incidence reported by BLNC in Bangladesh endemic unions was 2.62 malaria cases per 1000 population at risk from 2013 to 2016. As this represented 83% of the total malaria cases for 2013–2016, the overall incidence can be approximated to 3.2 cases per 1000. Thus the country could be classified as having “very low transmission” [21]. This would not, however, adequately characterize the spatiotemporal heterogeneity within the country. There is a need to identify and target areas with varying transmission levels in the affected areas to efficiently reduce, and eventually eliminate, the malaria burden [1]. Central to this is generation of higher temporal and spatial resolution data to ensure accuracy and effectiveness of interventions [1, 22]. The incidence of malaria in SeA was substantially higher than NeA. This has been shown previously [23–26]. Since the distribution of the 2007 Global Fund to Fight AIDS, Tuberculosis and Malaria grant, artemisinin-based combinations are free nationwide and the NMEP has achieved 100% LLIN coverage in SeA [1, 24, 26]. Notwithstanding, unions with a high malaria incidence persist in Khagrachhari, Bandarban and Rangamati. Targeting of interventions, such as vector control, is a key tenet of strategies for malaria elimination, which is the stated goal of the NMEP [27, 28]. High incidence unions are consistently located on international and/or district borders, towards northern Bandarban and southern Rangamati. While others have demonstrated that these districts have a high malaria incidence [1, 9, 23, 24], the results presented here are able to further define areas of relatively high malaria incidence within the districts.

From 2013 to 2016, a small proportion of the at-risk population experienced most malaria cases. In SeA, 11% of the population at risk accounted for 80% of malaria cases. In NeA, this relationship was 32/80%. Within districts with stable hotspots, the figures are remarkably similar to the 20/80 relationship described by Woolhouse et al. (for example, 21/80% for Rangamati and 19/80% for Netrakona) [16]. The present study is the first to demonstrate this relationship, nationwide, in Bangladesh. Ahmed et al. [4] found a similar relationship within two hypoendemic unions of Bandarban. Wangdi et al. [29] also reported this relationship in India, with malaria being concentrated in inaccessible, tribal and hilly areas. There is, therefore, spatial heterogeneity of malaria in Bangladesh; it is also equally important to consider the temporal clustering of cases.

Many investigators have documented the highly seasonal nature of malaria in SeA [4, 9, 30, 31]. The classically described high transmission season between May and October [24] is not evident in NeA. An explanation for this may be the rapidly declining incidence throughout NeA. Hu et al. [32] demonstrated that seasonality in incidence among five closely located villages differed markedly, with lower incidence villages showing a more erratic temporal distribution of cases. Zhang et al. [33] reported similar findings using China’s nationwide case data from 2004 to 2012, with seasonal peaks diminishing or disappearing as incidence declined. As an area nears elimination, heterogeneity in transmission is thought to increase; while there is clear spatial heterogeneity, there does not appear to be temporal heterogeneity in NeA.

There are several policy and practice implications of high incidence unions clustering on district and international borders. In- and out-migration is common in the CHTs and their unions [34]. Reports demonstrate the malaria risk of internally migrating groups to be elevated (e.g. traditional slash-and-burn, or jhum, cultivators) [4, 5, 35]. Thus there is a possibility that these internally migrating populations play a role in propagating malaria across district boundaries. Many of the stable union hotspots also share a border with Myanmar and/or India. Al-Amin et al. [36] showed that the species of vectors on international borders of Bangladesh are distinct from those that transmit malaria domestically. Thus, there may be a difference in the vector control strategies required to control *Anopheles* species mosquitoes at international border hotspots compared to domestically [37]. International malaria propagation can be attributed to short and medium distance population movement [38, 39], and cross-border migration is common across Bangladesh–Myanmar–India borders [27, 40]. For example, over 500,000 Rohingya refugees have fled from Myanmar to SeA of Bangladesh since 25 August 2017 [41]. There is evidence to show that artemisinin-resistant malaria parasites have spread north- and westward from the Thai-Myanmar border [42]. There are concerns that migrants and refugees from these areas may import antimalarial resistant parasites [1, 5, 26, 29, 43]. In the case that antimalarial resistance spreads to Bangladesh, it may be more likely to propagate further into India, the country with the largest at-risk population and highest number of cases of malaria in the SEA Region [29].

Throughout 2013–2016, there was hotspot stability in both space and time. When comparing years and seasons, a highly stable hotspot from one period reasonably predicted a future hotspot in the same union. Mogeni et al. [22] performed a meta-analysis of 19 African studies to assess the stability of micro-geographic hotspots over time. The authors found that, in four sites, hotspots of asymptomatic and febrile malaria were capable of predicting future clusters for up to 2 years, but not in the other 15 sites [22]. This comparatively poor predictive capacity is perhaps because of the different spatial scale at which the authors defined hotspots and/or that the hotspots were defined using point spatial processes, rather than discrete spatial analyses.

The present study was not designed to identify causal relationships of factors responsible for hotspot stability, however cautious inferences can be made. In SeA, the stable hotspots are in the most elevated and hilly sections to the southeast of the CHTs; they are densely forested with several streams running through them. Proximity to water bodies and forests are well-established malaria risk factors, especially in the CHTs [4, 15, 35, 44]. In NeA, stable hotspots are situated in sparsely vegetated plains south of the Indian border and West Khasi Hills. One of these hotspots is located in the Tanguar Haor, a wetland that is occupationally important for much of the surrounding population [45]. Seasonal agricultural workers and coal miners based in areas where transmission occurs have been suggested as the drivers of imported and introduced cases in these hotspots [5]. Therefore, social and demographic characteristics may confound approximations of environmental risk factors [15], as such further detailed study of these would be welcome additions to the literature on malaria risk in Bangladesh. NeA had relatively less stable hotspots for any given period, when compared to SeA. Mogeni et al. [22] described similar results in different settings. An explanation for lower hotspot stability is that spatial heterogeneity augments as malaria transmission declines. At a point, the statistical significance of hotspots becomes weaker due to smaller case numbers leading to reduced power. Thus, this may explain the relative instability of hotspots in NeA.

While spatial statistics have been used for malaria intervention planning in Bangladesh [46], this is the first instance of using hotspot stability maps to demonstrate malaria incidence hotspots over time. Haque et al. [1] used hotspot analysis to describe malaria prevalence in the endemic districts from 2008 to 2012. The authors found most hotspots for *P. falciparum* to be located within SeA. This analysis, however, was performed at a country level, which may not have allowed the stratification and identification of any hotspots within NeA. The hotspots for *P. vivax*, interestingly, were all located within NeA [1]. While not possible with the dataset available, hotspot analysis and stability maps done by species to a fine spatial and temporal resolution would be a welcome addition to this field of study.

Shasthya kormis are community health workers that perform malaria control and supervise community health volunteers; together, people in these two capacities perform the majority of direct malaria control activities in Bangladesh [7, 47]. The BLNC allocates shasthya kormis by union and all of their performance indicators are collected per union [7, 47]. Therefore, identifying and predicting hotspots at union-level is operationally valuable in this context. Bousema et al. [48] hypothesized that intensive malaria control targeted at hotspots could reduce transmission not only inside hotspots but also in adjacent areas. To test this, the authors conducted a cluster-randomized control trial in the Western Kenyan Highlands comparing intensive malaria interventions and standard interventions, both arms targeted at hotspots.

The primary outcome was parasite prevalence in zones outside hotspots. The trial demonstrated that the effect on prevalence was small, temporary and limited to targeted hotspot areas (and not surrounding areas) [48]. These findings suggest transmission may not primarily occur from hotspots to surrounding areas. This study’s analyses, however, lend themselves to further qualitative research to understand why populations in hotspots have higher malaria incidences over time, and operational research to understand context-specific best practice.

Hotspots at union-level do not appear to act consistently as sources of spread over time. There is evidence to show that hotspots identified at smaller spatial scales seed transmission and drive infection as a high transmission season progresses [12]. This has been demonstrated by showing that the same households within a village experience seasonal maximums of both mosquito and parasite prevalence, despite focal vector exposure in the dry season and widespread exposure in the wet season [12, 49]. Population movement within and outside of endemic unions occurs for a variety of reasons and as such, people may travel further away than the next union, making ‘contiguous’ spread from union to union less likely.

This study has several limitations. Data were not available on different species of malaria and it may be that the patterns of hotspots for *P. vivax* could differ from those for *P. falciparum*. This analysis was conducted at union level due to the level of aggregation of available data. A similar analysis at village level may enable more precise definition of hotspots, which could be targeted for elimination. This would require collection of village level malaria and population data and GPS coordinates. At the time of writing, there was no robust and representative means to adjust for net population growth at union level since last census data collection in 2011. The national annual growth rate since 2011 is estimated at around 1%. If this applied to the unions included in this analysis, the incidence would have been slightly overestimated. The surveillance system of Bangladesh is known to underreport infections; one reason being that private health services do not report malaria cases to the national register [25]. Additionally, not every case of malaria accesses care in a way in which they are recorded in the MIS, for example if a case were to buy over-the-counter anti-malarials from a pharmacy. There is however, no published nationwide information on the proportion of cases that seek alternative and private treatment in Bangladesh. The proportion of total malaria cases managed by the private sector (non-governmental, non-NGO) is thought to be an additional 1–10% on top of public sector cases (personal communication, NMEP). The micro-stratified dataset was from non-urban settings collected exclusively by the BLNC. This will have led to under-estimation of incidence rates as, for example, 17% of total cases in the CHTs were diagnosed by governmental healthcare workers from 2013 to 2016 (unpublished data, NMEP). Additionally, subclinical and submicroscopic infection not detected by routine surveillance play an important role in the malaria burden of Bangladesh, in terms of morbidity and infectious reservoir [35, 50, 51]. Finally, BRAC operates in non-urban settings where the incidence of malaria is likely to be higher than in other areas.

## Conclusions

The results of this study show that malaria in Bangladesh exhibited seasonal, hypoendemic transmission principally occurring in geographic hotspots and that these hotspots remained conserved over time. A small proportion of the population living in defined areas accounted for most malaria cases in Bangladesh. Stable hotspots had a modest predictive capability over the 4 years and this capability could be used to predict the occurrence of future hotspots. Having identified temporal and spatial clusters of malaria incidence, further studies are now required to understand the vector, sociodemographic and disease dynamics within these clusters. Given the low caseloads occurring in the low transmission seasons, and the conserved nature of malaria hotspots, these areas may be highly susceptible to interventions that interrupt transmission. Intense technical effort, political will and financial investment will be required as Bangladesh approaches its elimination target; directing more resources towards the identified areas may be an efficient way to achieve this target.
